# Carbon nanotube intramolecular p-i-n junction diodes with symmetric and asymmetric contacts

**DOI:** 10.1038/srep22203

**Published:** 2016-02-26

**Authors:** Changxin Chen, Chenghao Liao, Liangming Wei, Hanqing Zhong, Rong He, Qinran Liu, Xiaodong Liu, Yunfeng Lai, Chuanjuan Song, Tiening Jin, Yafei Zhang

**Affiliations:** 1 Key Laboratory for Thin Film and Microfabrication of the Ministry of Education, National Key Laboratory of Science and Technology on Micro/Nano Fabrication, Department of Micro/Nano Electronics, School of Electronic, Information and Electrical Engineering, Shanghai Jiao Tong University, Shanghai, 200240, China; 2 School of Physics and Information Engineering, Fuzhou University, Fuzhou, 350108, China

## Abstract

A p-i-n junction diode based on the selectively doped single-walled carbon nanotube (SWCNT) had been investigated, in which two opposite ends of individual SWCNT channel were doped into the p- and n-type SWCNT respectively while the middle segment of SWCNT was kept as the intrinsic. The symmetric and asymmetric contacts were used to fabricate the p-i-n junction diodes respectively and studied the effect of the contact on the device characteristics. It was shown that a low reverse saturation current of ~20 pA could be achieved by these both diodes. We found that the use of the asymmetric contact can effectively improve the performance of the p-i-n diode, with the rectification ratio enhanced from ~10^2^ for the device with the Au/Au symmetric contact to >10^3^ for the one with the Pd/Al asymmetric contact. The improvement of the device performance by the asymmetric-contact structure was attributed to the decrease of the effective Schottky-barrier height at the contacts under forward bias, increasing the forward current of the diode. The p-i-n diode with asymmetric contact also had a higher rectification ratio than its counterpart before doping the SWCNT channel, which is because that the p-i-n junction in the device decreased the reverse saturated current.

The diode is one of the most important basic components in integrated circuit. The excellent electrical and mechanical properties for the semiconducting single-walled carbon nanotube (SWCNT) afford it the ideal candidate material for preparing the diode^1^,^2^,^3^. The unique one-dimensional structure of SWCNTs enables it to be doped in specific segments of individual SWCNT to construct high quality of p-n junction^4^,^5^,^6^,^7^. The doping type and degree of the SWCNT can be controlled by non-covalent or adsorption doping without introducing any defects in SWCNTs to keep the excellent material characteristics of the SWCNTs^8^. The energy gap (Eg) of the SWCNT can be tuned by its diameter^9^ to fabricate the diodes with different built-in electric field. Besides, SWCNTs have extremely high intrinsic mobility^10^ (>100 000 cm^2^/Vs) and large current-carrying capacity^11^ as high as 10^9^ A cm^−2^. Therefore, the SWCNTis promising to be used for constructing high-performance diodes.

Some kinds of diodes based on SWCNTs had been investigated. The SWCNT Esaki diode was fabricated by using Kalium (K) to doped half of a SWCNT to get p-n junction^4^, but K caused many defects and had instability. A SWCNT p-i-n diode in which the carbon (C) atoms of the SWCNT were substitutionally doped by the boron (B) or nitrogen (N) atoms had been simulated and calculated by the non-equilibrium Green’s function method, showing negative differential resistance (NDR) effect in this kind of diode and rectification characteristics of the device depending on the different length of the intrinsic region in the SWCNT^5^. A p-i-n junction diode was fabricated by electrostatic doping in the two ends of the SWCNT using a split gate structure^6^,^7^. But, the diodes fabricated by the above mentioned methods were not unstable in the air or needed complex fabrication processes. For the Schottky barrier diode, the metals with different work functions were used as the contacts of the SWCNT to fabricate diode^12^,^13^,^14^. However, this kind of diode required an extra gate to realize the best rectification performance. Therefore, the good device structure design and optimized device process were desired to achieve high-performance SWCNT diode.

Here, a p-i-n junction diode based on the selectively doped single-walled carbon nanotube (SWCNT) had been investigated. In this kind of diode, two opposite ends of individual SWCNT channel were doped by triethyloxonium hexachloroantimonate (OA) and polyethylene imine (PEI) into the p- and n-type SWCNT respectively while the middle segment of SWCNT was kept as the intrinsic to result in the formation of p-i-n junction diode for good photovoltaic effect. The symmetric and asymmetric contact types were used to fabricate this diode respectively and studied the effect of the contact on the device characteristics. The symmetric-contact SWCNT p-i-n diode showed a rectification ratio of ~10^2^. The use of the asymmetric contact in the SWCNT p-i-n diode reduced the barrier between the metal electrode and SWCNT, causing the forward current increased by about one order of magnitude and a rectification ratio of >10^3^.

## Results and Discussion

**Figure 1:**
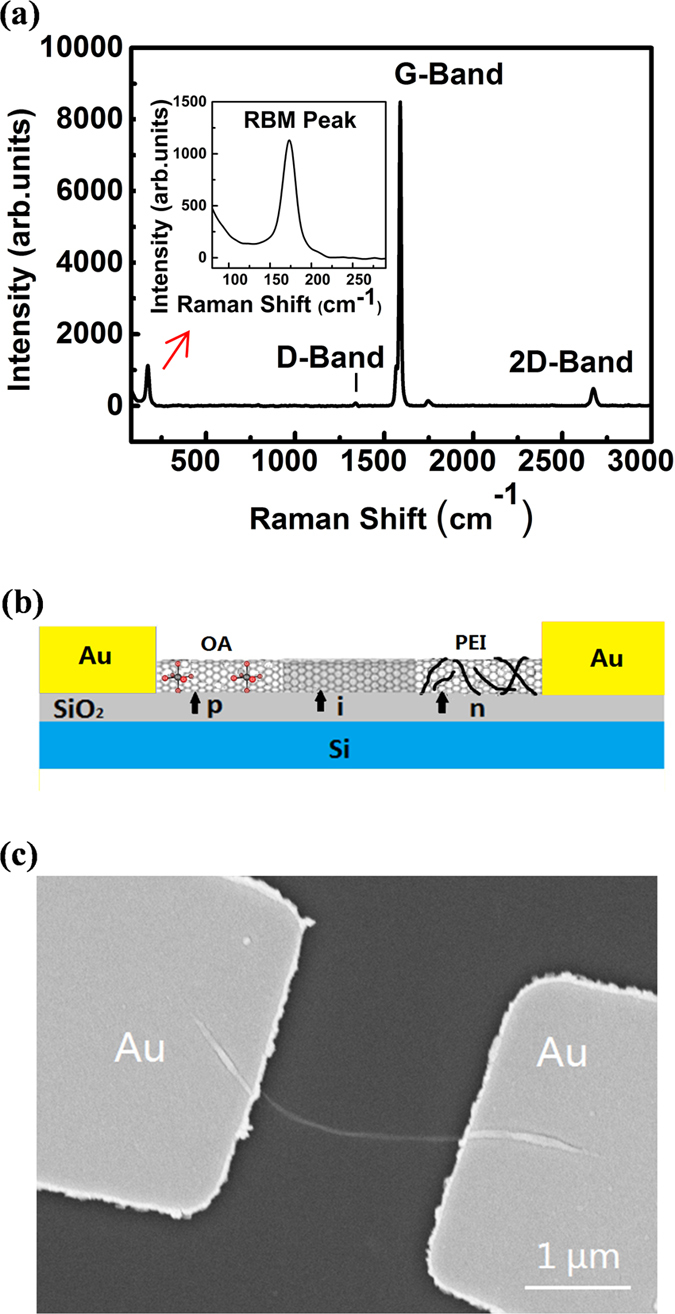
Illustration and characterization of the symmetric-contact SWCNT p-i-n diode. (**a**) Raman spectrum of the SWCNTs measured using a 532-nm laser as the excitation source. The inset shows the radial breathing mode of the SWCNTs in the low-frequency region. (**b**) Schematic diagram of the Au/Au contacted p-i-n SWCNT device. The length of each section (p, i, n) of the SWCNT was about 650 nm. The OA doped the left side of SWCNT to p-type. The PEI doped the right side of SWCNT to n-type. The middle area was protected by PMMA and kept intrinsic. (**c**) SEM image of the Au/SWCNT/Au device. The SWCNT channel length was ~2 μm.

The semiconducting SWCNTs (semiconductor purity ~ 99%) was used to fabricate the devices. The Raman spectra of the SWCNTs (Fig. 1a) was tested by the 532 nm laser. The SWNT diameters can be calculated to be ~1.4 nm according to the radial breathing mode (RBM) in the Raman spectrum (inset of Fig. 1a) using the following equation^15^.


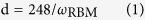


where ω_RBM_ is the wavenumber of the radial breathing mode in the Raman spectrum. The sharp G-band showed the semiconductor properties of the SWCNTs, and the G-band/D-band intensity ratio was more than two order of magnitudes, indicating that the number of defects in the SWCNTs was very small and graphitization degree of the SWCNTs was high.

The SWCNT p-i-n diode with symmetric contact (Fig. 1b) was fabricated and investigated. Firstly, the SWCNT aqueous solution was spun on the silicon (Si) chip with 100-nm-thickness thermally oxided SiO_2_ layer. The Au markers were fabricated on the Si chip for marking the location of the SWCNT. The suitable SWCNTs were selected out by atomic force microscope (AFM) or scanning electron microscope (SEM) for device fabrication. The Au-contacted SWCNT device with a channel length of ~2 μm (Fig. 1c) was fabricated by electron beam lithography and lift-off process (see Method for details). The OA^16^ and PEI^17^ were used to realize the n- and p-type doping of the SWCNT, respectively. The device was covered with polymethyl methacrylate (PMMA) and used e-beam lithography to provide a window on the left-side of the SWCNT. When the left 650 nm SWCNT exposed, the device was soaked in heated OA solution for one hour to finish the p-type doping. The same steps were performed to produce the window on the right side of the SWCNT (650 nm) and then the device was soaked in PEI solution for 8 hours to realize the n-type doping. After rinsing the extra unabsorbed PEI and PMMA by the methanol and acetone respectively, the Au-contacted SWCNT p-i-n diode prepared.

**Figure 2:**
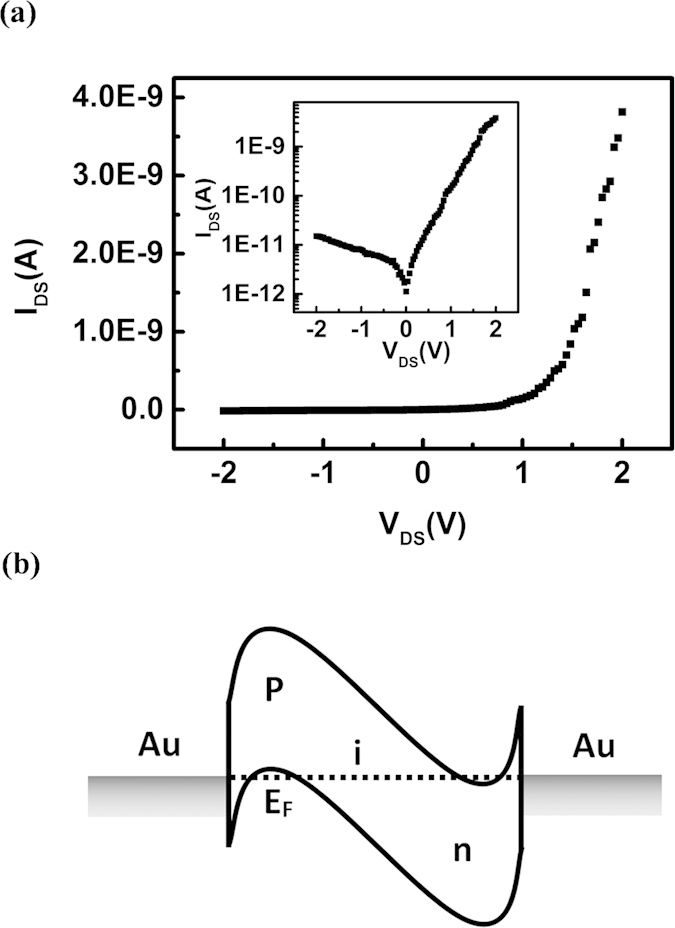
Rectification characteristics and energy band of the symmetric-contact SWCNT p-i-n diode. (**a**) I-V characteristics curve of the Au/Au symmetrically contacted p-i-n device. The inset showed the logarithmic curve. The reverse current was 15 pA, the forward current was 3.8 nA, and the rectification ratio was ~10^2^. (**b**) Energy band diagram for the device with Au as the drain (left) and the source (right). The Fermi level of Au was in the middle band gap of the SWCNT. The OA doped the SWCNT to p-type and the valence band bend upward to the Fermi level (dotted line). The PEI doped the SWCNT to n-type and the conduction band bend under the Fermi level.

The current-voltage (I-V) curve and the inset logarithmic curve (Fig. 2a) of the Au-contacted SWCNT p-i-n diode showed the reverse and forward currents were 15 pA and 3.8 nA, respectively The diode showed the typical rectifier characteristics and the rectification ratio was ~10^2^.

The reason for the rectification characteristics of the device was due to the p- and n-type doping of the OA and PEI on the two ends of the SWCNT channel respectively (Fig. 2b). The OA is one single-electron oxidant. When the OA reacted with the SWCNT, a stable charge-transfer complex between the SWCNT^+^ and SbCl_6_^−^ ion could be formed. The electrons would transfer from the SWCNT to the OA to increase the hole density in the SWCNT, resulting in the air-stable p-type doping of the SWCNT by the OA^16^. The PEI is one of the polymers containing the highest amine density. The long-chain PEI could entangle on the SWCNT to cause a non-covalent functionalization of the SWCNT, resulting in the air-stable n-type doping in the SWCNT^17^. Thus, a p-i-n junction could be formed by the doped p- and n-type regions and the undoped intrinsic region in the SWCNT.

**Figure 3:**
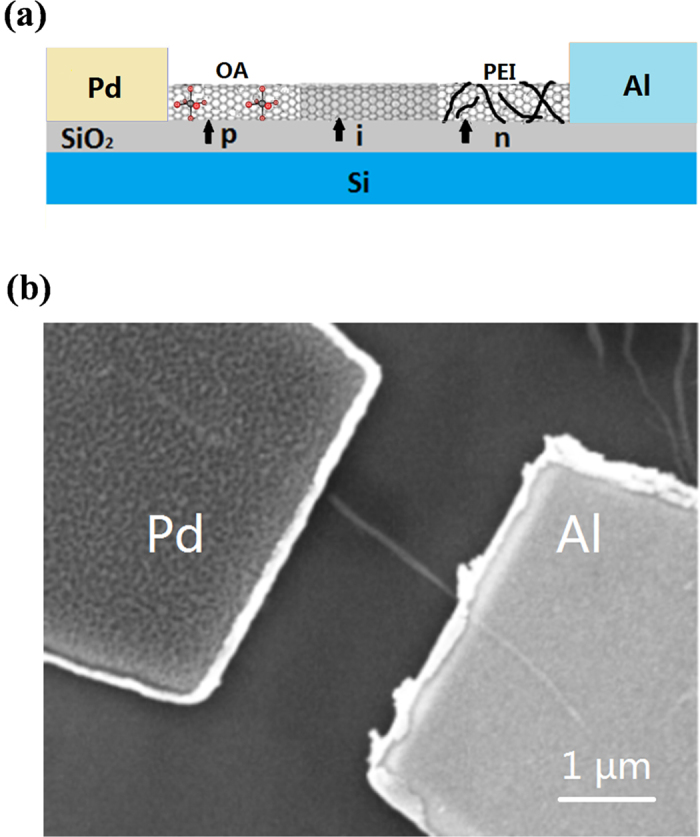
Illustration and characterization of the asymmetric-contact SWCNT p-i-n diode. (**a**) Schematic diagram of Pd/Al asymmetrically contacted p-i-n SWCNT device. Pd and Al were chosen as the drain and source, respectively. The window of PMMA near the Pd side was used for OA doping to obtain a p-type SWCNT and another one near the Al side was used for PEI doping to cause the SWCNT to become n-type. The middle area was always protected by PMMA and kept its intrinsic semiconductor properties. The length of each section (p, i, n) was about 650 nm. (**b**) SEM image of the Pd/SWNT/Al device. The left electrode is Pd and the right electrode is Al. The width of the channel is ~2 μm.

To study the effect of the contact, the SWCNT p-i-n junction diode with asymmetric contact was also fabricated and investigated by using the metals with different work function (Pd and Al) as the source and drain (Fig. 3a).

**Figure 4:**
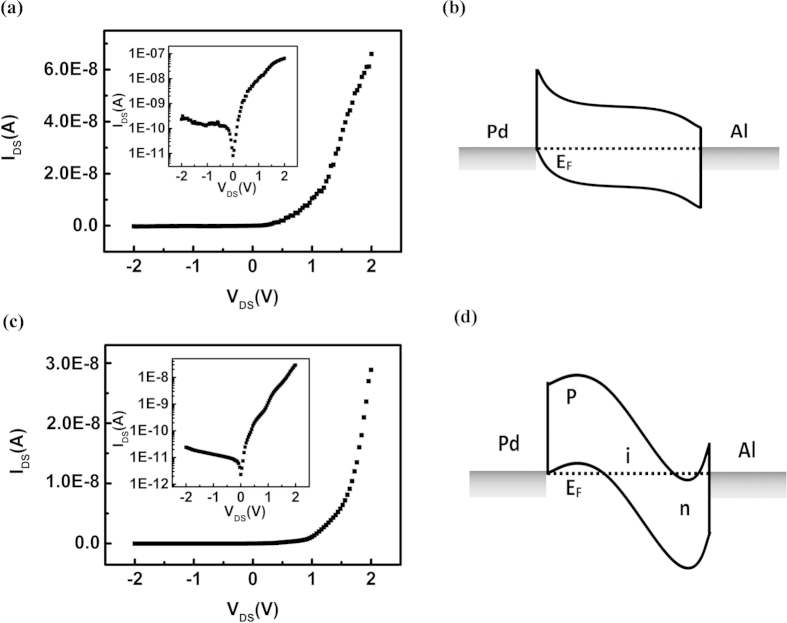
Rectification characteristics and energy band schematics of the asymmetric-contact SWCNT Schottky-junction diode before doping and the asymmetric-contact SWCNT p-i-n junction diode after doping. (**a**) I-V characteristic curves of asymmetrically contacted SWCNT-device. The inset shows the logarithmic curve, and the reverse and forward currents were 230 pA and 66 nA, respectively, and the rectification ratio was 2.9 × 10^2^. (**b**) Energy band diagram of asymmetrically contacted device. The Pd was the drain and had a high work function (5.1 eV) whose Fermi level was located at the bottom of the valence band of the SWCNT. The Al was the source and had a low work function (4.1 eV) whose Fermi level was located at the position that was a little lower than the bottom of the conduction band of the SWCNT. The dotted line represented the Fermi level and the band was in a state of balance. (**c**) I-V characteristic curves of the asymmetrically contacted p-i-n structure SWCNT device. The inset was the logarithmic curve. The reverse current for this device was 24 pA, the forward current was 29 nA, and the rectification ratio was ~10^3^. (**d**) Energy band diagram of the asymmetrically contacted partition-doped p-i-n structure device. The OA on the SWCNT near the Pd electrode caused the SWCNT segment to become p-type and the energy band of that SWCNT segment to bend upward; The PEI on the SWCNT near the Al electrode caused the SWCNT segment to become n-type and the energy band of that SWCNT segment to bend downward.

This p-i-n junction diode with asymmetric contact was constructed with a 1.4-nm-diameter semiconducting SWCNT. Firstly, the Pd and Al electrodes were patterned on two ends of the SWCNT respectively by the e-beam lithography and lift-off technique to form a SWCNT channel length of 2 μm (see Method for details). The obtained device showed a behavior of the Schottky junction diodes^8^. The dark I-V characteristics was shown in Fig. 4a. When the positive bias was applied, the channel current was about 66 nA. Conversely, the reverse current was about 230 pA. The rectification ratio was ~300. The rectification characteristic of the asymmetric-contact structure could be explained as follows (Fig. 4b). The work function of Pd was 5.1 eV, the Fermi level of Pd would be located at the top of the valence band of SWCNT at the Pd-SWCNT contact. The work function of Al was 4.1 eV, the Fermi level of Al would be close to the bottom of the conduction band of SWCNT at the Al-SWCNT contact^9^. The difference in the values of the work functions of the source and drain electrodes caused the bending of the energy band in the SWCNT and made the device showed the diode characteristics.

Then, the resulted asymmetric-contact structure was doped with OA and PEI by the same manner as that for symmetric-contact device (see Method for details). The device characteristics of the asymmetric-contact SWCNT p-i-n diode was tested, showing a rectification ratio of 1.2 × 10^3^. The rectification ratio was significantly higher than the Au/Au contacted SWCNT p-i-n diode or the Pd/Al contacted Schottky diode. The reverse current of this device was 24 pA, the forward current was 29 nA (Fig. 4c). By comparison, this reverse current was lower than the Pd/Al contacted structure one order of magnitude, the forward current was higher than the Au/Au contacted p-i-n diode one order of magnitude.

As mentioned above, the use of the Pd and Al as the electrodes reduced the Schottky barrier between the metal and SWCNT and caused the increase of the forward current of the diode for the asymmetric-contact SWCNT p-i-n diode. The characteristics was explained by the energy band diagram in Fig. 4b. When a positive bias was applied, the electrons need to overcome the Schottky barrier between Al and the SWCNT first and then can flow into the channel, but this barrier was lower than the barrier between Au and the SWCNT. On the side of Pd, without the barrier, holes flowed into the p-type area easily. Consequently, positive bias caused carriers to overcome the Pd/SWCNT barrier easily and gave rise to a large injection current and made the forward current much higher than the Au/Au symmetric-contact p-i-n device. The doping made the barrier between the p- and n-type areas higher than the barrier between the metal and SWCNT. When a negative bias was forced, carriers were blocked by the Schottky barrier and the p-i-n junction, the reverse current.was mainly controlled by built-in electric field in the SWCNT. The reverse current was almost the same as for the Au/Au symmetric-contact p-i-n device. This explained the higher rectifying performance of this device.

In summary, a p-i-n junction diode based on the selectively doped SWCNT had been investigated. In this kind of device, two opposite ends of individual SWCNT channel were doped into the p- and n-type SWCNT respectively while the middle segment of SWCNT was kept as the intrinsic to cause the formation of the p-i-n junction with a strong built-in electric field. The symmetric and asymmetric contacts were used to fabricate the diodes respectively. Both kinds of diodes showed the typical rectification characteristics. A low reverse saturation current of ~20 pA could be achieved by them. It was found that the use of the asymmetric contact can effectively improve the performance of the p-i-n diode, with the rectification ratio increased from ~10^2^ for the device with the symmetric contact to >10^3^ for the one with the asymmetric contact. The asymmetrically-contacted SWCNT p-i-n junction diode with a high rectification ratio is promising to be applied as good photovoltaic device or be used in next-generation integrated circuit.

## Methods

### Fabrication of the devices with symmetric and asymmetric contacts

The silicon (Si) wafer with an thermally-oxided SiO_2_ layer (~100 nm) were used for fabricate the diodes. The Au cross markers was fabricated on the wafer by the optical lithography (SUSS MA6/BA6 Double-sided UV Mask Aligner) and the lift-off technique. The SWCNT aqueous solution which a concentration of about 2.5 ng/ml (NanoIntegris Inc.) was used in our experiment, in which the purity of semiconducting SWCNTs was about 99% and the average diameter of the SWCNTs was about 1.4 nm. Disperse SWCNTs were obtained on the Si chips by spinning the SWCNT solution at 1200 rpm for 2 minutes. The resulted Si chips were rinsed in the de-ion water and isopropal (IPA) to remove the surfactant on the spun Si chips and then dried at 80 °C for a couple of minutes. Then, the suitable SWCNTs with their locations marked by the Au cross markers were selected out for device fabrication. The 950k PMMA was spun on the silicon chips at 1500 r.p.m. for 30 seconds and followed drying the resist at 180 °C for 20 minutes to form an about 300-nm-thickness PMMA film covering the SWCNTs. The electrodes of device was fabricated by an e-beam lithography system (DY-2000A) and a magnetron sputtering system (Ulvac Ultra-high Vacuum Sputtering System, AEMD). The SWCNT channel between the Au electrodes (100 nm) was 2 μm. For the Pd (100 nm)/Al (100 nm) contacted device, the SWCNT channel was 2 μm and two applications of e-beam lithography and magnetron sputtering were performed. When the electrodes were completed, the devices was underwent with the annealing treatment for 20 minutes at a temperature of 200 °C and vacuum pressure of 5 × 10^−3^ Pa.

### Selectively doped of SWCNTs in the diodes

The triethyloxonium hexachloroantimonate (OA, Sigma Aldrich) was used to p-type doping,. The OA solution was produced by dissolving 50 mg of OA in 10 mL of methanol. The device was covered PMMA (300 nm) on surface and the e-beam lithography system was used to provide the window (0.65 μm × 2 μm) on left side of the SWCNT, then soaked the device in the OA solution for 1 h at 70 °C. Methanol was used to clean the device and acetone was used to clean the PMMA and drying in the air. The polyethylene imine (PEI, average molecular weight ~25000, Sigma Aldrich) was used to n-type doping. The PEI solution was produced by dissolving the PEI in methanol with density about 10 wt%. The device with the window (0.65 μm × 2 μm) on right side was soaked in the PEI solution for 8 hours at room temperature. The device was washed with methanol and the PMMA was cleaned by acetone and drying in the air.

### Characterization and electrical measurements

The Raman spectra (Fig. 1(a)) of the SWCNTs were tested by Dispersive Raman Microscope (Senterra R200-L) by using a 532-nm laser as the excitation source. The SEM images of the Au/SWCNT/Au device (Fig. 1(c)) and Pd/SWCNT/Al device (Fig. 3(b)) were obtained using a Zeiss ultra 55 scanning electron microscope at 5 kV of acceleration voltage. The I-V curve of the device was tested by an Agilent 4156C semiconductor parameter meter instrument. For the test, the OA-doped side of the electrode (Au or Pd) was the drain and the PEI-doped side (Au or Al) was the source. All I-V characteristics were performed at dark condition and without the gate voltage, the V_DS_ sweep was from −2.0 V to 2.0 V.

## Additional Information

**How to cite this article**: Chen, C. *et al.* Carbon nanotube intramolecular p-i-n junction diodes with symmetric and asymmetric contacts. *Sci. Rep.* **6**, 22203; doi: 10.1038/srep22203 (2016).
